# Post-Traumatic Ostial Avulsion of a Polar Inferior Renal Artery Treated by Endovascular Covered Aortic Stenting

**DOI:** 10.5334/jbsr.2081

**Published:** 2020-05-07

**Authors:** François Carrozza, Fabrice C. Deprez

**Affiliations:** 1 Department of Diagnostic and Interventional Radiology, CHU UCL Namur, Université catholique de Louvain, BE

**Keywords:** blunt aortic trauma, accessory renal artery, endovascular treatment, c-arm CBCT

## Abstract

Renovascular traumas are rare in abdominal blunt traumas, especially those involving complete avulsion of a renal artery. Their management poses a dilemma between blood flow preservation and the risks of bleeding. We present the case of a rare variant of renovascular injury, with a post traumatic ostial avulsion of a polar inferior renal artery, successfully treated percutaneously by endovascular aortic covered stenting under c-arm cone-beam computed tomography guiding.

## Introduction

Renal trauma accounts for approximately 3% of all trauma admissions [1], the vast majority (80–90%) of cases resulting from blunt rather than penetrating injury. Post-traumatic renovascular lesions are even more rare (0.08% of all abdominal blunt traumas) [2,3]. In case of serious renal injuries, other organs are frequently injured (75–80%) [2].

Approximately 15–30% of all adult patients have at least one renal accessory artery. Management of injuries to these arteries poses a dilemma between invasiveness, functional loss, blood flow preservation, and the risks of bleeding. We herein present the case of a rare variant of renovascular injury, with an ostial avulsion of a polar inferior artery following blunt trauma that was successfully managed by a covert aortic stenting. No report of this kind was found in the literature.

## Case Report

**Figure 1:**
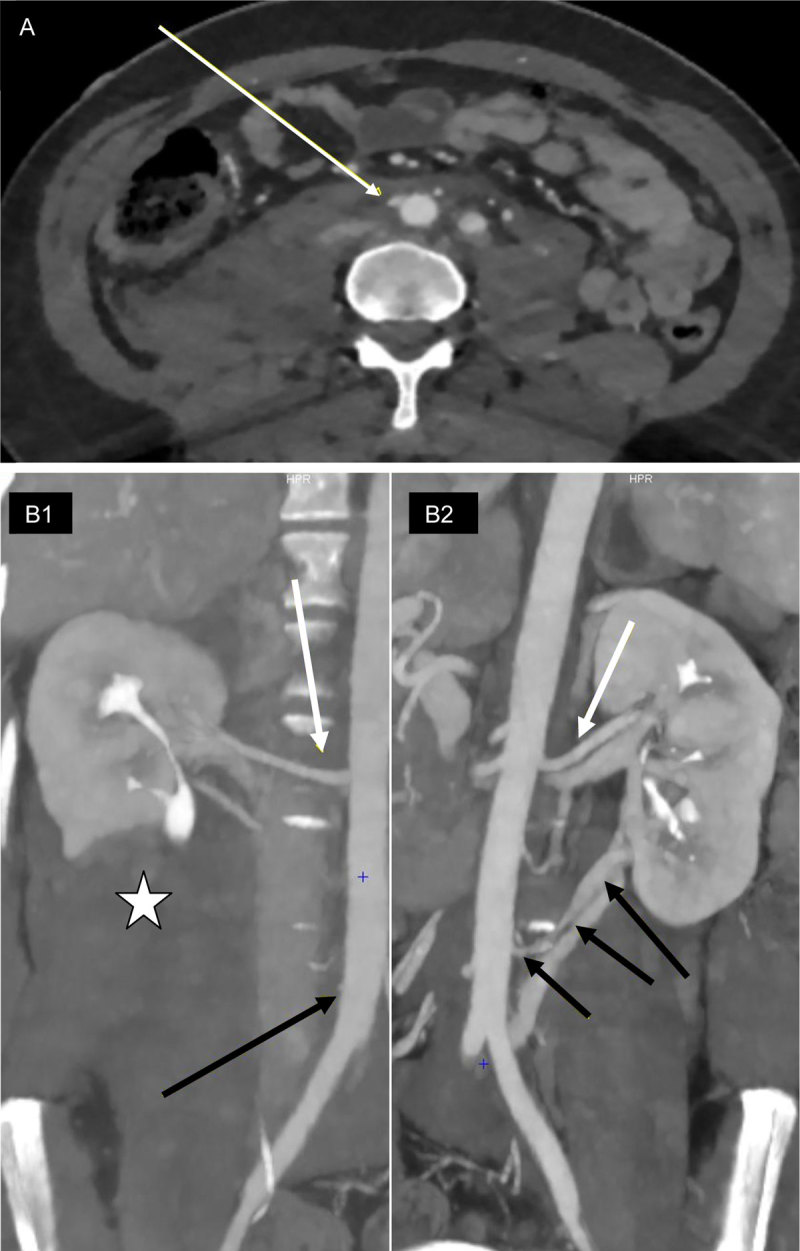
**A:** Enhanced axial CT of the aorta, with an ulcer-like contour (arrow) corresponding to ostial avulsion of the right polar inferior artery (RPIA). **B:** Oblique coronal views of the right (B1) and the left (B2) arterial vascularization of the kidneys, with two main renal arteries (white arrows), a left polar inferior artery (LPIA – B2, black arrows) and avulsion of the RPIA (B1, black arrow) associated to an infarction of the lower pole of the right kidney (white star).

A 34-old-woman without any medical history was admitted after high-speed motorcycle accident. The patient was hemodynamically stable. A multiphase post-contrast total-body computed tomography (CT) revealed several fractures (burst fracture of T4, left scapula, sacrum and several right lumbar transverses processes), pulmonary contusion and avulsion of the right polar inferior artery (RPIA). The RPIA was located 2 cm above the iliac bifurcation, at the same level as the inferior mesenteric artery (IMA). The avulsion was at the ostium of the artery, showing an ulcer-like contour of the aorta. A retroperitoneal hematoma was associated, but with no active bleeding, meaning a spontaneous thrombosis of the RPIA had occurred. There was no enhancement of the lower pole of the right kidney, corresponding to an infarcted segment supplied by the RPIA (Figure 1). A contralateral (left side) polar inferior artery (LPIA) was also present.

**Figure 2:**
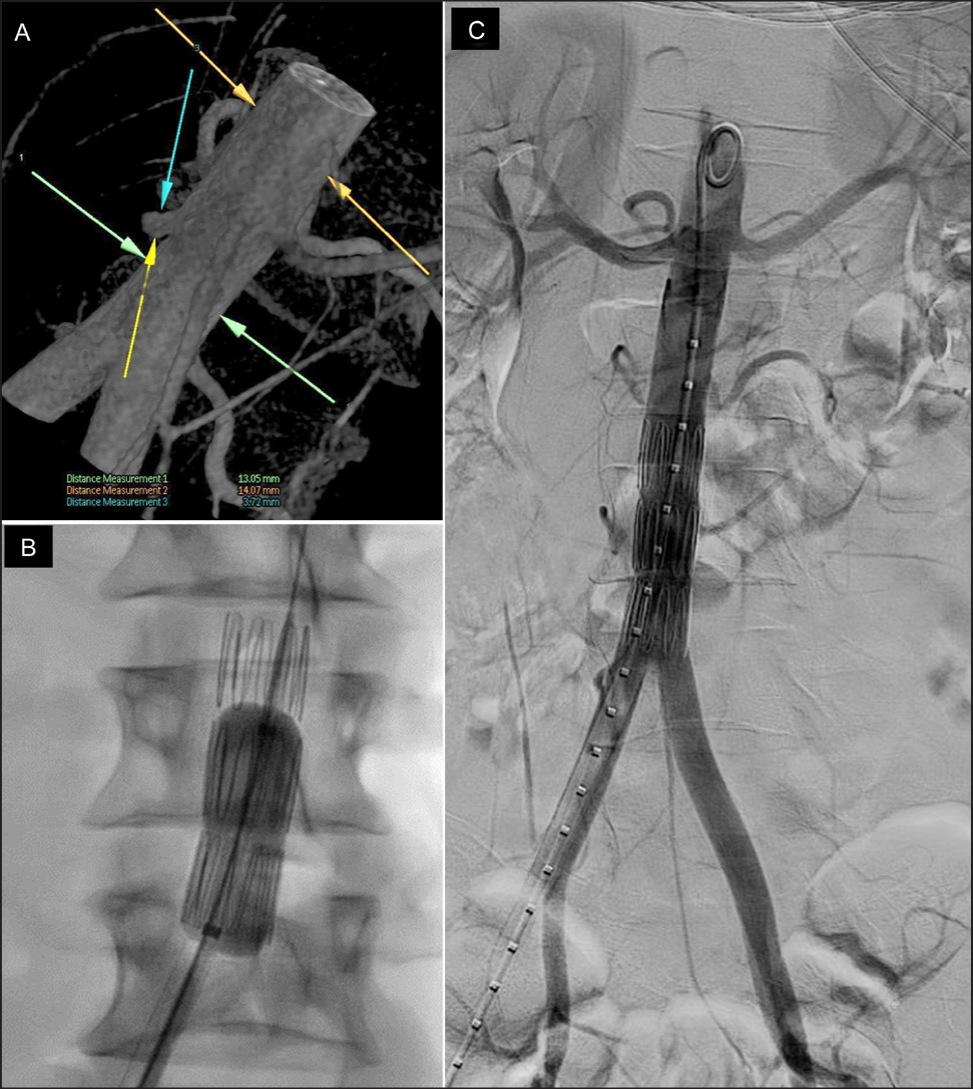
**A:** 3D(VR) c-arm cone-beam CT view of the aorta, for calibrated measurements, 3D-roadmap and planning of the stenting. The proximal avulsion of the RPIA looks like a 3.7 mm pseudo-aneurysm of the aorta. **B:** 18 × 56 mm self-expandable stent with 14-mm balloon remodeling. **C:** Final angiogram showing perfect positioning of the stent, covering both RPIA and LPIA, associated with non-enhancement of the lower pole of both left and right kidney.

Considering the high risk of rebleeding from the aortic stump, we decided to undergo interventional radiology procedure under local anesthesia, using a c-arm CBCT with 3D road-map guidance. The ostial stump of the RPIA was too small to be embolized (4 × 6 mm), with a high risk of perforation. Therefore, we opted for a covered stent. Due to the narrow diameter of the aorta (14 mm), we couldn’t use conventional aorto-iliac bifurcated stent graft, so we decided to use a self-expandable stent graft (Zilver 18 × 56 mm, Cook Medical) usually used for iliac extension during endovascular aortic repair (EVAR). This was the shortest one available at the time. A balloon-expandable covered stent was not available either. The inferior mesenteric artery and the left inferior renal artery were inevitably covered, with no consequences for the IMA and an expected infarction of the lower third of the left kidney. The angiographic post-procedure control was excellent (Figure 2). Vascular closure device was used (Perclose ProGlide, Abbott Vascular) after 12 Fr vascular sheath removal. Immediate follow-up was without complication. CT performed one year after the treatment confirmed total patency of the aortic stent graft, cortical atrophy of the lower pole of both kidneys and a retrograde perfusion of the IMA. Patient’s blood pressure (120/80 mm Hg) and renal function were normal.

## Discussion

Complete avulsion of the renal artery is a rare but life-threatening injury. CT reveals infarction of the kidney associated with extensive medial perirenal hematoma. Active bleeding on the proximal end of the tear may also be seen [2].

In our case, an accessory renal artery (ARA) was involved, not the main renal artery. Moreover, the lesion was located very close to the ostium of the RIPA. It could be considered as grade 4 renal trauma, corresponding to a segmental renal artery or vein injury (noted by a segmental parenchymal infarct or main renal artery or vein injury with contained hematoma) [4,5] and as a blunt aortic pseudo-aneurysm.

Complications of blunt abdominal aortic injuries are severe, and non-operative management cannot be considered due to high mortality risk (75%) [6]. The treatment of pseudoaneurysms in blunt aortic trauma is either surgical or endovascular (stent graft placement). Open surgical treatment with direct aortic suture of the avulsion is a very invasive approach, with a high rate of morbidity, and it involves an unsightly skin scar (to be avoided especially in young female patients). As in our case, patients hemodynamically stable with pseudoaneurysm and with a high likehood of survival from other associated injuries can be considered for semi-selective (<1 week) repair [7]. Endovascular repair with commercially available stent grafts can safely and effectively treat blunt abdominal aortic injury.

Percutaneous access is associated with a lower frequency of groin infection or lymphocele, and a shorter procedure time and hospital length of stay compared with open surgery. The use of conscious sedation with local anesthesia and percutaneous femoral access has further decreased the morbidity of procedure [8]. Complications of endovascular repair include further aortic wall injury, access complications and contrast-induced nephropathy, but this treatment is associated with less perioperative morbidity and mortality [6]. In our case, the use of endovascular procedure also includes the covering of the LPIA, increasing the risk of associated renal function loss.

Greenberg et al. reported that accessory renal arteries generally perfuse discrete parenchymal areas and are end arteries [9]. In most cases of their study, accessory renal arteries coverage during EVAR don’t result in compromised renal function or worsening hypertension, even in patients with accessory renal arteries larger than 3 mm in diameter. These results were confirmed by Karmacharya et al. and Malgor et al., confirming that intentional occlusion of accessory renal arteries during EVAR is safe and without consequence [10,11].

## Conclusion

Post-traumatic ostial avulsion of an accessory right polar inferior renal artery is an extremely rare but life-threatening situation that can be successfully treated by endovascular stent-graft placement. Partial renal infarction due to traumatic avulsion or intentional endovascular covering of an ARA is not associated with renal failure or increased hypertension.
